# Tele-education to improve residents’ knowledge and quality of care in hospital hyperglycemia: a multicenter randomized clinical trial

**DOI:** 10.20945/2359-4292-2026-0041

**Published:** 2026-04-20

**Authors:** Jivago da Fonseca Lopes, Gabryela Mariane Costa e Silva, Conrado Reinoldes Caetano, Raissa Rigon Riboli, Emanoela Gomes Pinheiro, Bárbara Luíza Petter Bortoluzzi, Gustavo Dutra Leite Nunes, Maiane Siewes de Souza, Maria Carolina Wensing Herdt, Aline Scharnberg, Carolina Garcia Soares Leães Rech, Leticia Schwerz Weinert, Maristela Bohlke

**Affiliations:** 1 Universidade Federal de Pelotas, Departamento de Medicina Interna, Pelotas, RS, Brasil; 2 Universidade Católica de Pelotas, Curso de Medicina, Pelotas, RS, Brasil; 3 Universidade Federal do Rio Grande, Escola de Medicina, Rio Grande, RS, Brasil; 4 Universidade Católica de Pelotas, Internato em Clínica Médica, Pelotas, RS, Brasil; 5 Universidade Federal de Pelotas, Internato em Clínica Médica, Pelotas, RS, Brasil; 6 Universidade Federal de Ciências da Saúde de Porto Alegre, Escola de Medicina, Porto Alegre, RS, Brasil; 7 Universidade Federal do Rio Grande, Hospital Universitário Dr. Miguel Riet Corrêa Jr., Rio Grande, RS, Brasil; 8 Universidade Federal de Ciências da Saúde de Porto Alegre, Internato em Clínica Médica, Porto Alegre, RS, Brasil; 9 Universidade Católica de Pelotas, Internato em Endocrinologia, Pelotas, RS, Brasil; 10 Universidade Católica de Pelotas, Departamento de Endocrinologia, Pelotas, RS, Brasil; 11 Universidade Federal de Pelotas, Internato em Endocrinologia, Pelotas, RS, Brasil; 12 Universidade Católica de Pelotas, Programa de Pós-Graduação em Saúde e Comportamento, Pelotas, RS, Brasil

**Keywords:** Medical education, hospital hyperglycemia, diabetes mellitus, telemedicine

## Abstract

**Objective:**

To evaluate the impact of a structured tele-education program on hospital hyperglycemia and diabetes, focusing on residents’ medical knowledge and inpatient care.

**Subjects and methods:**

This open-label, multicenter, randomized clinical trial enrolled internal medicine residents from four university hospitals in southern Brazil. Teams were block-randomized to an intervention group that received an online lecture plus 30 days of tele-education via WhatsApp, or to a control group with no intervention. The primary outcome was medical knowledge, assessed with a validated 10-item questionnaire. Secondary outcomes included quality of insulin prescriptions, hypoglycemia and hyperglycemia rates, and hospital length of stay (LOS). Analyses were performed using SPSS v29 (5% significance).

**Results:**

Fifty residents completed the study. The intervention group achieved higher post-intervention knowledge scores than the control group (median 8 vs. 6 correct answers; p = 0.005) and showed significant improvement from preto post-test (6 to 8; p < 0.001), with consistent gains across centers. Clinical data from 149 hospitalized patients were analyzed (mean age 67.8 years; 55% female); 56% had diabetes, and 44% had hospital-related hyperglycemia. There was a nonsignificant trend toward more appropriate NPH (p = 0.107) and regular insulin (p = 0.203) prescriptions in the intervention group. Median LOS was longer in the intervention group (19 vs. 13 days; p = 0.009).

**Conclusion:**

The tele-education program improved residents’ knowledge of inpatient hyperglycemia. Larger studies are needed to confirm clinical effects and long-term outcomes of tele-education in hospital glycemic management.

## INTRODUCTION

Diabetes is a complex chronic condition that requires continuous medical care, multifactorial risk reduction, and appropriate glycemic control. In the hospital setting, dysglycemia - articularly hospital hyperglycemia - is common and is associated with higher risks of complications and prolonged length of stay (LOS) ^(1)^.

In Brazil, data from the Brazilian Longitudinal Study of Adult Health (ELSA-Brasil) - a cohort of 15,103 civil servants aged 35 to 74 years from six state capitals - reported a diabetes prevalence close to 20%, with approximately half of the cases previously undiagnosed ^(2)^.

In the United States, an estimated 22% of hospitalized patients have diabetes, and hospitalizations account for nearly half of the US$174 billion annual medical expenditures related to the disease. Each year, approximately 1.6 million new cases are diagnosed, with an overall prevalence of 23.6 million (7.8% of the population), of which about one quarter are undiagnosed ^(3)^.

Hospital readmissions are frequent among people with diabetes, with admission rates ranging from 14% to 20% ^(4)^, especially within 30 days of discharge ^(5)^. Major risk factors for readmission include lower socioeconomic status, race/ethnicity, comorbid conditions, and recent hospitalization ^(4)^.

Although no single protocol universally prevents readmissions, targeted strategies for high-risk patients and transitional care models have demonstrated benefits ^(6-9)^. Glycemic disturbances in hospitalized patients - hyperglycemia, hypoglycemia, and glucose variability - have been consistently linked to adverse outcomes, including increased morbidity and mortality ^(10)^.

Clinical evidence also underscores the role of physician knowledge and specialized diabetes teams in predicting shorter hospital stays and improved outcomes ^(11-13)^.

Scalable educational approaches may help close these gaps. Tele-enabled formats - including remote monitoring, messaging platforms, and decision support - show promise, though most studies focus on outpatients or primary care rather than inpatient settings ^(14-17)^.

Given the clinical burden of hospital hyperglycemia, rising costs, and readmission risk, there is a need to evaluate pragmatic educational strategies for clinicians. This study investigates whether a tele-education program - comprising an online lecture and WhatsApp-based microlearning - can improve internal medicine residents’ knowledge of inpatient hyperglycemia and influence prescribing practices and the management of glycemic extremes during hospitalization.

## SUBJECTS AND METHODS

This was an open-label, multicenter, randomized clinical trial conducted with internal medicine residents from four teaching hospitals in southern Brazil: the University Hospital of the Federal University of Pelotas (HE-UFPel), the University Hospital Dr. Miguel Riet Corrêa Jr. of the Federal University of Rio Grande (HU-FURG), the University Hospital São Francisco de Paula of the Catholic University of Pelotas (HUSFP-UCPel), and the *Santa Casa de Misericórdia de Porto Alegre*, a teaching hospital affiliated with the Federal University of Health Sciences of Porto Alegre (UFCSPA).

Clinical teams, rather than individual residents, were randomized in blocks to minimize contamination between participants. Randomization was performed using WinPepi software (version 11.65), with stratification by center to ensure balanced allocation across the participating hospitals.

All internal medicine residents from clinical teams at the four institutions were invited to participate. At the time of the study, 51 residents were eligible. Exclusion criteria included being under endocrinological supervision, being on vacation during the intervention period, or declining participation. Of those invited, 50 completed the preand post-intervention assessments.

Residents allocated to the intervention group received a live online theoretical lecture, followed by a one-month tele-education program on hospital hyperglycemia delivered via WhatsApp. The program consisted of short videos and supporting materials prepared by three endocrinologists. Videos were shared multiple times, and residents were asked at the end of the intervention to confirm that they had watched the content, ensuring full exposure to the materials. Participants were also able to submit questions during the intervention period. The control group did not receive the lecture or any additional educational support and continued with routine clinical practice.

Medical knowledge regarding inpatient hyperglycemia was assessed in both groups before and after the intervention using a 10-item questionnaire with content validity evidence ^(13)^. This baseline assessment served to control for residents’ prior knowledge levels and to enable within-group comparisons before and after the intervention. Each correct answer was scored as 1 point, for a maximum of 10 points.

In a second phase, clinical data were collected retrospectively from medical records of patients hospitalized under the care of participating residents during the intervention month. Data collection occurred after patient discharge and included demographics, comorbidities, prescriptions, and glycemic outcomes. The educational program was strictly educational and noninterventional in patient care.

The primary outcome was the improvement in residents’ knowledge scores. Secondary outcomes were quality of inpatient care indicators, including appropriate prescription of capillary blood glucose monitoring based on nutritional route, use of oral antihyperglycemic agents in the hospital, and insulin therapy practices - particularly the use of NPH and correctional regular insulin. Additional outcomes were the frequency of hyperglycemia (capillary glucose > 180 mg/dL), hypoglycemia (< 70 mg/dL), and hospital LOS. Prescriptions from the last 5 days of hospitalization were reviewed by trained researchers using standardized procedures. Prescriptions were considered appropriate if they followed international recommendations (American Diabetes Association and Endocrine Society) and national guidelines from the Brazilian Diabetes Society on screening and management of hospital hyperglycemia in noncritical patients ^(18)^.

Dichotomous variables were described as absolute and relative frequencies (n, %). Continuous variables were presented as mean and standard deviation, or median and interquartile range (IQR) when non-normally distributed. Normality was assessed using the Shapiro-Wilk test. Between-group comparisons were performed using the Student’s *t* test for normally distributed data and the Mann-Whitney U test for nonparametric data. Categorical variables were compared using Pearson’s chi-square test or Fisher’s exact test, as appropriate. Logistic regression models were used to control for potential confounders and identify independent predictors, including variables with clinical or statistical relevance (p < 0.20) in univariate analysis. A significance level of 5% was adopted. Analyses were conducted using IBM SPSS Statistics, version 29.0 (IBM Corp., Armonk, NY, USA).

Sample size estimation was based on previous findings of low baseline knowledge regarding inpatient hyperglycemia, with an average accuracy of 46.4% in a pilot study ^(13)^. Assuming an improvement to ~80% correct responses after the intervention, a significance level of 5%, and statistical power of 80%, the minimum required sample size was 62 completed questionnaires.

Participation was voluntary, with written informed consent obtained from all residents. The questionnaire was applied confidentially, and clarifications were provided as needed. Patient data were collected only with institutional authorization, following formal approval for medical record use. Confidentiality and privacy, particularly for sensitive institutional information, were strictly maintained.

The study protocol was approved by the local research ethics committee (approval number 7.393.105) and registered at ClinicalTrials.gov (identifier NCT07108426).

## RESULTS

A total of 51 internal medicine residents were invited to participate: 15 (30%) from UFPel, 7 (14%) from UCPel, 7 (14%) from FURG, and 21 (42%) from UFCSPA. Of these, 50 residents completed both the preand post-intervention questionnaires, while one declined participation.

**Figure 1:**
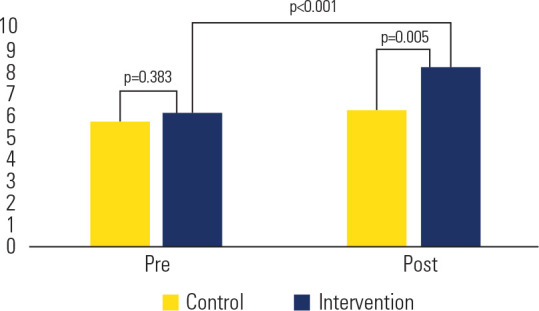
Improvement in medical knowledge scores between groups before and after the intervention, evaluated using a validated questionnaire comprising 10 questions on hospital hyperglycemia.

Before the intervention, the median number of correct answers was 5.5 (IQR 5-6) in the control group and 6 (IQR 5-7) in the intervention group (p = 0.383). After the intervention, knowledge scores were significantly higher in the intervention group compared with the control group (median 8 [IQR 8-9] vs. 6 [IQR 5-8]; p = 0.005). Within-group analysis showed an improvement of 2.5 points in the intervention group from preto post-test (p < 0.001). The control group also demonstrated a modest but significant improvement (median 5.5 [IQR 5-7] to 6 [IQR 5-8]; p = 0.009) (**Figure 1**). Subgroup analysis by center confirmed consistent knowledge gains across all participating hospitals (p < 0.05 for each).

Following the educational intervention, 149 patient medical records were reviewed: 55 (36.9%) from UFPel, 31 (20.8%) from UCPel, 30 (20.1%) from FURG, and 33 (22.1%) from UFCSPA. Patients had a mean age of 67.8 ± 15.6 years, and 82 (55%) were female. Among them, 84 (56%) had a previous diagnosis of diabetes, with a median disease duration of 9.5 years (IQR 2-15 years). The remaining 65 patients (44%) were classified as having hospital-related hyperglycemia. None of the patients had type 1 diabetes. The most common reasons for hospitalization in general were infectious diseases (34.9%), cerebrovascular conditions (16.8%), and neoplasia (14.8%), followed by renal and hepatic diseases (7.4% and 6.7%, respectively). Other less frequent causes accounted for 13.4% of admissions. The most common comorbidity was cerebrovascular disease, present in 103 patients (69.1%). **Table 1** summarizes the baseline characteristics of patients by study group.

**Table 1:** Clinical and demographic characteristics of hospitalized patients, by group (control vs. intervention)

Variable	Control (n = 73)	Intervention (n = 76)	P value
Age, years	69.5 ± 13.8	66.2 ± 17.2	0.2
Male sex, n	33 (45.2)	34 (44.7)	0.954
Diabetes duration, years	6.5 (2-14)	10 (6-20)	0.422
HbA1c	6.3 (5.75-8.75)	6.6 (5.9-9.2)	0.806
Type 2 diabetes, n	43 (58.9)	41 (53.9)	0.542

Data are shown as n (%), mean (standard deviation), or median (interquartile range).

HbA1c: glycated hemoglobin.

Analysis of clinical outcomes is presented in **Table 2**. There was a nonsignificant trend toward more appropriate use of insulin in the intervention group (NPH insulin, p = 0.107; regular insulin prescribed based on isolated capillary glucose, p = 0.203). However, median hospital LOS was longer in the intervention group compared with the control group (19 days [IQR 11-39 days] vs. 13 days [IQR 7-25 days], respectively; p = 0.009). In exploratory analyses, the only factor significantly associated with prolonged LOS was the study center. The longest stays were observed at UFCSPA (median 26 days [IQR 14.5-46.5 days]) and UFPel (median 24 days [IQR 13-37 days]), while UCPel (median 13 days [IQR 6.5-18 days]) and FURG (median 7 days [IQR 6-11 days]) had shorter stays (p < 0.001).

**Table 2:** Care indicators related to the management of hospital hyperglycemia, by group (control vs. intervention)

Indicator	Control (n = 73)	Intervention (n = 76)	P value
Blood glucose at admission, n	48 (65.8)	51 (67.1)	0.861
Correct prescription, n			
Metformin	7 (9.6)	8 (10.5)	0.685
NPH insulin	48 (65.8)	59 (77.6)	0.107
Regular insulin	55 (75.3)	59 (77.6)	0.742
Sliding scale insulin	51 (69.9)	60 (78.9)	0.203
HbA1c at admission, n	24 (32.9)	27 (35.5)	0.733
Appropriate glucose monitoring, n			
Oral diet	46 (63)	48 (63.2)	0.985
Enteral tube feeding	13 (17.8)	6 (7.9)	0.07
Total parenteral nutrition	0	1 (1.3)	0.325
Nothing by mouth	1 (1.4)	0	0.306
Hyperglycemia, n	56 (76.7)	52 (68.4)	0.257
Hypoglycemia, n	4 (5.5)	7 (9.2)	0.534
Hospital stay, days	13 (7-25)	19 (11-39)	0.009

Data are shown as n (%) or median (interquartile range).

## DISCUSSION

This multicenter randomized trial demonstrated that a structured educational intervention on hospital hyperglycemia, delivered via tele-education, significantly improved internal medicine residents’ knowledge across four teaching hospitals. These results reinforce the importance of continuous medical education strategies aimed at enhancing physicians’ understanding of inpatient glycemic management. The remote design - an online lecture combined with 1 month of support via WhatsApp, a widely available and free platform - illustrates the feasibility of implementing scalable, low-cost educational programs across diverse training contexts.

The consistent improvement observed across all participating hospitals suggests that such interventions may be applicable to different institutional settings. This finding is supported by previous evidence. A meta-analysis of randomized clinical trials reported that telemedicine interventions, particularly those involving feedback and two-way communication, were effective in improving glycemic control, resulting in significant reductions in HbA1c levels. ^(15)^ Our study adds to this body of knowledge by demonstrating similar benefits in the inpatient context, targeting the education of residents rather than direct patient management.

High adherence, with only one resident declining participation, underscores the acceptability and practicality of WhatsApp as an educational tool. This is particularly relevant for health systems facing resource constraints or geographic barriers, where accessible platforms are critical to ensure continuity of medical education.

Our previous observational study on medical education in hospital hyperglycemia found comparable improvements, though without the methodological rigor of randomization ^(13)^. Similarly, Desimone and cols. ^(17)^ conducted a randomized trial with residents and reported significant knowledge gains in the management of corticosteroid-induced and perioperative hyperglycemia, though without clear improvements in clinical outcomes. Together, these findings suggest that while education reliably enhances physician competence, translation into measurable patient outcomes likely requires additional strategies. Moreover, translating individual knowledge into effective clinical practice is inherently complex. Factors such as institutional culture, competing clinical priorities, and limited feedback mechanisms may hinder behavior change, despite educational improvement.

Analysis of patient records in our study indicated a nonsignificant trend toward more appropriate use of NPH insulin and correctional regular insulin prescriptions in the intervention group. This suggests a potential influence of the educational program on prescribing practices, although knowledge gains did not fully translate into consistent behavior change. Similar challenges have been described in multicenter studies of resident physicians, which identified gaps in confidence and familiarity with insulin use and institutional protocols ^(14)^. These data highlight the importance of structured ongoing education combined with supervised clinical support to achieve more robust improvements in care quality and patient safety.

An unexpected finding was the longer hospital LOS observed in the intervention group. One possible explanation is a learning-curve effect, in which residents exposed to the intervention sought to optimize glycemic control more rigorously, inadvertently prolonging hospitalization. Another contributing factor may be institutional heterogeneity, as substantial variation in LOS was observed across centers, likely reflecting differences in workflows, case complexity, and resource availability. In addition, the high prevalence of cerebrovascular disease among patients may have contributed to these differences, as this comorbidity is often associated with longer hospitalizations and greater clinical complexity. Although our sample size was limited, this factor likely reflects the real-world profile of the inpatient population across centers. Previous studies have shown that patients with diabetes are more prone to prolonged or repeated hospitalizations, not only due to disease severity but also because of systemic factors in care coordination ^(6)^. Future research should examine whether the longer LOS is transient and improves as residents gain experience and institutional systems adapt.

This study has limitations. The sample size of patients was modest, follow-up was short, and knowledge retention over time was not assessed. Verification of residents’ exposure to educational materials relied on self-report, and the internal consistency of the short knowledge questionnaire was moderate. Qualitative evaluation of participants’ perceptions was not performed, and cost-effectiveness or feasibility analyses were not included. In addition, standardized severity indices were not collected across centers, which limits further adjustment for baseline clinical complexity. Nevertheless, residents’ prior knowledge was controlled through the pre-test and post-test design, ensuring comparable baseline levels between groups. Despite these limitations, strengths include the randomized multicenter design - rare in medical education studies focused on inpatient glycemic control - and the use of a pragmatic, low-cost tele-education platform. By evaluating both knowledge and clinical outcomes, this trial provides a more integrated assessment of educational interventions in the hospital setting.

In conclusion, the structured tele-education intervention significantly improved internal medicine residents’ knowledge on the management of hospital hyperglycemia, with consistent effects across multiple teaching hospitals. While trends toward better prescribing practices were observed, further research with larger samples and longer follow-up is required to determine whether such educational strategies translate into sustained improvements in clinical outcomes.

## Data Availability

datasets related to this article will be available upon request to the corresponding author.

## References

[1] American Diabetes Association Professional Practice
Committee (2024). 16. Diabetes care in the hospital: standards of care in
diabetes-2024. Diabetes Care.

[2] Schmidt MI, Hoffmann JF, Diniz MFS, Lotufo PA, Griep RH, Bensenor IM (2014). High prevalence of diabetes and intermediate hyperglycemia: the
Brazilian Longitudinal Study of Adult Health (ELSA-Brasil). Diabetol Metab Syndr.

[3] Moghissi ES, Korytkowski MT, DiNardo M, Einhorn D, Hellman R, Hirsch IB (2009). American Association of Clinical Endocrinologists and American
Diabetes Association consensus statement on inpatient glycemic
control. Diabetes Care.

[4] Rubin DJ. (2015). Hospital readmission of patients with diabetes. Curr Diab Rep.

[5] Bansal V, Mottalib A, Pawar TK, Abbasakoor N, Chuang E, Chaudhry A (2018). Inpatient diabetes management by specialized diabetes team versus
primary service team in non-critical care units: impact on 30-day
readmission rate and hospital cost. BMJ Open Diabetes Res Care.

[6] Jiang HJ, Stryer D, Friedman B, Andrews R. (2003). Multiple hospitalizations for patients with
diabetes. Diabetes Care.

[7] Maldonado MR, D’Amico S, Rodriguez L, Iyer DP, Balasubramanyam A. (2003). Improved outcomes in indigent patients with ketosis-prone
diabetes: effect of a dedicated diabetes treatment unit. Endocr Pract.

[8] Wu EQ, Zhou S, Yu A, Lu M, Sharma H, Gill J (2012). Outcomes associated with post-discharge insulin continuity in US
patients with type 2 diabetes mellitus initiating insulin in the
hospital. Hosp Pract (1995).

[9] Hirschman KB, Bixby MB. (2014). Transitions in care from the hospital to home for patients with
diabetes. Diabetes Spectr.

[10] Umpierrez G, Cardona S, Pasquel F, Jacobs S, Peng L, Unigwe M (2015). Randomized controlled trial of intensive versus conservative
glucose control in patients undergoing coronary artery bypass graft surgery:
GLUCO-CABG trial. Diabetes Care.

[11] Umpierrez GE, Reyes D, Smiley D, Hermayer K, Khan A, Olson DE (2014). Hospital discharge algorithm based on admission HbA1c for the
management of patients with type 2 diabetes. Diabetes Care.

[12] Mendez CE, Umpierrez GE. (2014). Pharmacotherapy for hyperglycemia in noncritically ill
hospitalized patients. Diabetes Spectr.

[13] Lopes JF, Andrade PDR, Borges MT, Krause MC, Simi MOS, Bohlke M (2024). Medical education on hospital hyperglycemia improving knowledge
and outcomes. Arch Endocrinol Metab.

[14] Horton WB, Law S, Darji M, Conaway MR, Akbashev MY, Kubiak NT (2019). A multicenter study evaluating perceptions and knowledge of
inpatient glycemic control among resident physicians: analyzing themes to
inform and improve care. Endocr Pract.

[15] Faruque LI, Wiebe N, Ehteshami-Afshar A, Liu Y, Dianati-Maleki N, Hemmelgarn BR (2017). Effect of telemedicine on glycated hemoglobin in diabetes: a
systematic review and meta-analysis of randomized trials. CMAJ.

[16] Bergenstal RM, Layne JE, Zisser H, Gabbay RA, Barleen NA, Lee AA (2021). Remote application and use of real-time continuous glucose
monitoring by adults with type 2 diabetes in a virtual diabetes
clinic. Diabetes Technol Ther.

[17] Desimone ME, Blank GE, Virji M, Donihi A, DiNardo M, Simak DM (2012). Effect of an educational inpatient diabetes management program on
medical resident knowledge and measures of glycemic control: a randomized
controlled trial. Endocr Pract.

[18] Marino EC, Momesso D, Toyoshima MTK, de Almeida MFO, Schaan BD, Negretto LAF (2025). Screening and management of hospital hyperglycemia in
non-critical patients: a position statement from the Brazilian Diabetes
Society (SBD). Diabetol Metab Syndr.

